# Cast Extruded Films Based on Polyhydroxyalkanoate/Poly(lactic acid) Blend with Herbal Extracts Hybridized with Zinc Oxide

**DOI:** 10.3390/polym16141954

**Published:** 2024-07-09

**Authors:** Magdalena Zdanowicz, Małgorzata Mizielińska, Agnieszka Kowalczyk

**Affiliations:** 1 Center of Bioimmobilisation and Innovative Packaging Materials, Faculty of Food Sciences and Fisheries, West Pomeranian University of Technology, Szczecin, Janickiego 35, 71-270 Szczecin, Poland; mmizielinska@zut.edu.pl; 2 Faculty of Chemical Technology and Engineering, West Pomeranian University of Technology, Szczecin, Piastów Ave. 42, 71-065 Szczecin, Poland; agnieszka.kowalczyk@zut.edu.pl

**Keywords:** biocomposites, *Chelidonium* L., *Hypericum* L., PHB, polyhydroxyalkanoates, poly(hydroxybutyrate) herbal extracts, *Urtica* L., zinc oxide

## Abstract

The aim of the presented work was to functionalize a blend based on polyhydroxyalkanoate (PHA): poly(hydroxybutyrate (PHB) with poly(lactic acid) (PLA) and a mixture of three selected herb extracts, namely, *Hypericum* L., *Urtica* L. and *Chelidonium* L., (E), zinc oxide (ZnO) and a combined system (EZnO), produced via extrusion. Before processing with bioresin, the natural modifiers were characterized using thermal analysis, FTIR and antimicrobial tests. The results revealed interactions between the extracts and the filler, leading to higher thermal stability in EZnO than when using E alone. Moreover, the mixture of extracts exhibited antimicrobial properties toward both Gram-negative (*S. aureus*) as well as Gram-positive bacteria (*E. coli*). Modified regranulates were transformed into films by cast extrusion. The influence of the additives on thermal (DSC, TGA and OIT), mechanical, barrier (WVTR and OTR), morphological (FTIR) and optical properties was investigated. The EZnO additive had the highest impact on the mechanical, barrier (OTR and WVTR) and optical properties of the bioresin. The microbial test results revealed that PHA-EZnO exhibited higher activity than PHA-ZnO and PHA-E and also reduced the number of *S. aureus*, *E. coli* and *C. albicans* cells. The findings confirmed the synergistic effect between the additive components. Modified polyester films did not eliminate the phi6 bacteriophage particles completely, but they did decrease their number, confirming moderate antiviral effectiveness.

## 1. Introduction

The usage of disposable plastics, which occur mainly in the food packaging industry but are also used in applications such as healthcare, is a topic being discussed worldwide, and efforts are being made to eliminate the impact of these plastics on the environment. One of the methods of plastic waste management is to go back to the material’s design and the use of biodegradable polymers. According to European Commission data, the 10 most commonly found single-use plastic items on European beaches represent 70% of all marine litter in the EU [1]. To fight plastic pollution, the EU created the Single-Use Plastic Directive (SUP) [2]. One of the goals of implementing the regulations included in the document is to reduce the use of single-use plastic products and replace them with compostable non-petroleum-based materials. These implementations can be observed nowadays, e.g., when some products made with paper or plastics with “compostable” logos are more widely available. The most common bio-degradable plastic is poly(lactic acid). However, due to its origin (it is produced from biomass via polycondensation, via a synthetic process), conforming with the directive requirements is problematic. Poly(hydroxyalkanoate)s—PHA—are a group of polyesters produced by various microorganisms. These biopolyesters can be formed with ca. 150 hydroxyalkanoates with an average molecular weight in the range of 5 × 10^4^–2 × 10^7^ Da [3]. PHAs are thermoplastic polymers that can be processed via the conventional methods typical for plastics (e.g., injection molding and extrusion). Their properties (processability, mechanical, barrier and thermal properties) are dependent on microbial species, growth conditions and the type of monomeric unit [4]. The advantages of PHAs are their hydrophobicity, high quality of barrier against gases, compostability and non-toxicity. However, due to the high cost of production, the industrialization of PHA is limited [3]. One of the methods of using PHA-based final products is blending them with other polymers, especially with poly(lactide acid) (PLA) [5]. The properties of post-synthetized PHA-based materials can be altered by the addition of fillers, plasticizers or other additives. The introduced components not only affect the physico-chemical properties but also bring some extra functionality, such as antimicrobial properties. For example, the introduction of terpenes into the polymer matrix can not only plasticize polyester but also give it some antimicrobial properties [6,7], which are especially useful in food packaging or medical applications. Terpenes are highly volatile, with an intense fragrance that can affect the organoleptic properties of the food; they are thermally non-stable and can be irritative to the skin. Thus, novel green functionalizers for biopolyesters are being sought and studied.

Many extracts of *Hypericum* species, *Chelidonium* L. and *Urtica* L. have been investigated as antimicrobial agents against many bacterial strains [8,9,10,11,12,13,14]. It was demonstrated that active compounds such as hypericine, pseudohypericine, xanthous (from the *Hypericum* herb) [8], chelidonine, sanguinarine, berberine, chelerythrine, coptisine and/or protopine), flavonoids, phenolic acids (from *Chelidonium* L.) [10,11,12], flavonoids (rutinosides of quercetin, glucosides, isorhamnetin and kaempferol), caffeoyl-esters (chlorogenic acid, neochlorogenic acid), scopoletin (coumarin), caffeic acid, sitosterol besides polysaccharides, fatty acids, lignans, lectin and ceramides were biologically active. Additionally, twelve compounds belonging to terpenes: α-pinene, limonene, γ-terpinene, β-pinene, geraniol, linalool, eucalyptol, camphor, α-terpineol, carvacrol, methyl chavicol and eugenol (from *Urtica* L.) [13,14] were confirmed to have antimicrobial, anti-inflammatory, antiviral, antitumor, antioxidant, immunomodulatory, choleretic, analgesic, hepatoprotective, cardiovascular, antiulcer, hepatoprotective, diuretic and vasoconstrictive properties. Ethanolic extracts from *Hypericum* demonstrated strong effectiveness against Gram-positive *Bacillus cereus* and *Staphylococcus aureus* strains, as well as against Gram-negative *Escherichia coli* and *Pseudomonas aeruginosa* cells [8,9]. *Chelidonium* L. extracts were found to be active against Gram-positive bacterial strains such as *S. aureus* (including MRSA strains), *S. epidermidis*, *Streptococcus mutans*, *B. cereus* and fungal (*Fusarium oxysporum* and *Botrytis cinerea*) and yeast strains (*Candida albicans*) [10,11,12]. Moreover, several researchers demonstrated the antiviral, antibacterial (against *E. coli*, *Listeria monocytogenes*, *Salmonella enterica*, *Bacillus subtilis*, *Micrococcus luteus* and *Serratia marcescens*) and antifungal activity of *Urtica* L. extracts. It is worth mentioning that a cooperative effect between *Urtica* L. extracts and other active agents such as antibiotics was noted [14,15,16]. Previous studies carried out in our research unit revealed that the activity of these plant extracts can be boosted with the addition of ZnO, although the studies were related mainly to the coating materials [17,18,19]. In the literature, there are no studies related to PHA-based films using combined systems with herbal extracts in nanoparticles, while some works presented the use of PLA with plant-origin additives [20]. Only a few works report some results for PHA. Fuente-Arias et al. prepared PHBV films and phenolic compounds via compression molding [21], while Latos-Brozio and Masek prepared PLA/PHB extruded films with plant-based pigments [22]. This work, for the first time in the literature, presents a preliminary study of the influence of the mixture of herbal extracts and the herbal mixture including ZnO nanoparticles in the bioresin matrix.

The purpose of this work was the functionalization of poly(hydroxyalkanoate)-based material (PHA) (commercial blend with PLA) with green naturally originated active agents prepared from the three selected herbal mixtures of *Hypericum* L., *Chelidonium* L. and *Urtica* L. or a mixture of extracts modified with nano-zinc oxide (ZnO), intended for food packaging and healthcare applications. The mixture of different herbs, instead of a single herb, was used to broaden their activity toward different microbiological strains. The films were obtained via cast extrusion. The physico-chemical properties (i.e., mechanical properties, barrier oxygen transmission rate (OTR), water transmission rate (WVTR), optical (UV-Vis and color on the CIELab scale) and thermal properties (differential scanning calorimetry (DSC) and thermogravimetric analysis (TGA)), as well as the morphology (FTIR spectroscopy and SEM) of neat PHA with modified films were compared. Moreover, microbiological studies, including antimicrobial, antifungal and antiviral tests, were carried out. The basic tests (TGA, FTIR and microbiological tests) for the active agents based on the extracts used were also carried out.

## 2. Materials and Methods

### 2.1. Materials

A polymer matrix bioresin, based on semi-crystalline, biodegradable, biopolyester poly(hydroxybutyrate)–P(3HB-*co*-4HB) (3HB:4HB = 13:1) [23] with the addition of PLA and some additives (Ecomann PHA EM40000 from Shenzhen Ecomann Biotechnology Co., Ltd., Guangdong, China), was used in this study. The modifier compositions were formed with Atmer110^TM^, which is an ethoxylated sorbitan ester (31% biobased, CRODA, Chocques, France) that is used as a liquid carrier for herbal extracts and nanofiller. The mixture of extracts was prepared with *Hypericum* L., *Urtica* L. (Kawon, Gostyń, Poland) and *Chelidonium* L. (Farmvit, UK). Zinc oxide (ZnO) was obtained from Permedia Colors (Lublin, Poland).

The analysis of antimicrobial (antibacterial and antiviral) properties was conducted using selected bacteria, such as Gram-positive *Bacillus subtilis* DSMZ 1090 and *Staphylococcus aureus* DSMZ 346 strains and Gram-negative *Escherichia coli* DSMZ 498, *Pseudomonas syringae* van Hall 1902 DSM 21482 (this strain was used to analyze antibacterial activity and as a phi6 phage host) and Φ6 bacteriophage DSM-21518 (used as a SARS-CoV2 surrogate to investigate antiviral activity). The microorganisms and phi6 phage were purchased from the Leibniz Institute Deutsche Sammlung von Mikroorganismen und Zellkulturen (DSMZ, Braunschweig, Germany). The antifungal effectiveness investigation was carried out using the yeast strain *C. albicans* PCM 2566 (purchased from the Polish Collection of Microorganisms).

Ethanol (98%, Warchem, Trakt Brzeski, Poland) was used for the extraction of the herbs. Additionally, dimethyl sulfoxide—DMSO (Warchem, Trakt Brzeski, Poland) was used to prepare herb extracts for preliminary studies on their antimicrobial activity. To determine the antimicrobial properties of the modifier systems, MacConkey agar, TSA (tryptic soya agar), acting as a medium, and Luria-Bertani (LB) and TSB broths (Merck, Darmstadt, Germany) were used. All media were suspended in 1 L of distilled water and sterilized in an autoclave (at 121 °C for 15 min).

### 2.2. Preparation of the Additives

At first, the plant extracts were prepared separately using selected plants: *Hypericum* L., *Urtica* L. and *Chelidonium* L. An amount of 150 g of each dry herb was introduced separately into 900 mL of 98% ethanol. Then, the ethanolic systems (in sealed bottles) were placed in a microwave (Amica, Wronki, Poland) for 15 min at 70 °C and then in a shaker (Ika, Staufen im Breisgau, Germany) for 1 h at 70 °C (150 rpm). The extraction was accomplished according to the procedures described by the authors [24,25,26], with slight modifications. The solid residue of the herbs was separated from the extracts using a Büchner funnel. Then, 300 mL of each of 3 individual extracts were mixed together to obtain 900 mL of extract mixture. In the next step, 10 g of Atmer was added to 300 mL of the ethanolic mixture of 3 extracts to prepare the E additive. The EZnO additive was obtained via mixing 300 mL of the ethanolic extracts with 10 g of Atmer and 0.3 g of ZnO nanoparticles. The rest of the herbal mixture was used for the preliminary study of its antimicrobial properties (S1 in the Supplementary Materials). The systems were left for ethanol evaporation (at 55 °C) to obtain dark-green-colored viscous final additives. Table 1 presents the component amounts, calculated as the weight part per 100 parts of bioresin (pph).

### 2.3. Film Preparation

In the first step, the neat, dried bioresin granulate was mixed by hand with the prepared additives (1.33 pph of Atmer per 100 pph of bioresin + 0.04 pph ZnO, extracts or extracts + ZnO) (see Table 1) and extruded with a co-rotating twin-screw extruder (L/D = 40) at a profile temperature of 130/135/145 × 8 °C at 60 rpm. Then, the regranulate was cast-extruded with a flat die (width of the film, ca. 17 cm) on a LabTech line (L/D = 30) at the profile temperature of the extruder (140/150/155/155 °C, co-extruder 160 °C and die 160 °C, chill roll 50 °C). The bioresin processing was performed using a LabTech line (Labtech Engineering Co., Ltd., Phraeksa, Thailand).

### 2.4. Characterization of the Additives and the Biopolymer Films

FTIR spectrophotometric analysis was performed with a Perkin Elmer spectrophotometer (Spectrum 100, Waltham, MA, USA) equipped for the ATR technique. The spectra were analyzed in 32 scans in a wavenumber range of 4000–600 cm^−1^ using Omnic software. The thermal properties (DSC and TGA) of E and ZnO, as well as of the films, were studied using differential scanning calorimetry (DSC 250, TA Instruments, New Castle, DE, USA) for determination of the phase transitions of the samples (ca. 10 mg), which were analyzed in hermetic aluminum pans at a temperature range of −50–200 °C (heating rate of 10 °C/min). Two DSC measurements for each composition were carried out. Thermogravimetric analysis (TGA) was performed using a Q5000 thermoanalyzer (TA Instruments, New Castle, DE, USA). Small doses (ca. 10 mg) of the samples were examined in an air atmosphere. The temperature range was 20 ÷ 900 °C, with a heating rate of 10 °C/min. Oxidation induction time (OIT) tests were determined with the DSC 250 (TA Instruments, USA), according to the ISO11357-6 [27]. A sample (5 ± 0.5 mg) was heated, in standard aluminum pans under nitrogen flow (50 mL/min) at a rate of 20 °C/min, from room temperature to the set OIT temperature (250 °C), then recorded isothermally under air flow (50 mL/min) for 60 min. The heat flow was recorded in isothermal conditions as a function of time. The characteristic oxidation peak parameters were determined, i.e., the time to oxidation peak (maximum) and end set.

Tensile testing of the polyester films (without additives or modified films) was performed using a Zwick/Roell (Ulm, Germany) machine equipped with a 2.5 kN load cell on 15-millimeter-width strips of samples, according to the method described in previous work [26]. The grip separation was 50 mm, and the testing speed was 100 mm/min. The test was carried out for at least 6 repetitions for each specimen in ambient conditions. The tests were performed in the machine direction.

The film surface was examined using a scanning electron microscope (SEM). As a first step, the samples were placed on pin stubs and coated with a thin layer of gold in a sputter coater at 24 °C (Quorum Technologies Q150R S, Laughton, East Sussex, UK). As the next step, SEM micrographs were taken using a Vega 3 LMU microscope (Tescan, Brno-Kohoutovice, Czech Republic). The microscopic analysis was carried out using a tungsten filament with an accelerating voltage of 10 kV.

For an examination of barrier properties regarding oxygen, the moisture oxygen transmission rate (OTR) was determined according to the standard ASTM D3985 [28] with the OXTRAN model 2/20 (Mocon, Minneapolis, MN, USA) at RH 0% and 23 °C, while the water vapor transmission rate (WVTR) was measured according to the standard ASTM F1249-20 [29] with PERMATRAN 3/33 (Mocon, Minneapolis, MN, USA) at RH 100% and compensation at 90%. The measurements were carried out on a 5 cm^2^ sample area at 37.8 °C.

Optical properties, including CIELab color scale measurements, were determined with a CR-5 colorimeter (Konica Minolta, Tokyo, Japan). Samples were analyzed in 5 repetitions at random locations on each studied sample. Yellowness index (YI), chroma (*c) and color changes (∆E*) were calculated according to the equations given in [18]. Unmodified PHA film was used as a reference. The analysis was repeated after 1 week of storage in ambient conditions for non-covered samples. The UV-Vis spectrophotometer UV-Vis Thermo Scientific Evolution 220 (Waltham, MA, USA) was employed to analyze the transparency (T, transmittance at 700 nm) and UV-ray absorption capacity in the wavelength range of 190–900 nm.

The PHA and functionalized films were cut into squares (30 × 30 mm). Then, the antimicrobial effectiveness of the PHA, PHA-ZnO, PHA-E and PHA-EZnO films was determined according to the ASTM E 2180-01 standard [30]. To analyze the antiviral activity of the PHA films and PHA film modified with the active agents, Φ6 phage lysate was purified according to the method reported by Bhetwal et al. [31]. Then, the Φ6 lysate was prepared according to the method presented by Bonilla et al. [32]. The antiviral activity of the active films was compared to the pure PHA films (control samples) and was examined according to a modified ISO 22196-2011 standard [28]. Finally, Φ6 bacteriophage particle amplification was carried out, as suggested by Skaradzińska et al. [33]. To examine the host (*P. syringae*) cultivation rate in real time after its incubation/contact with the PHA, PHA-ZnO, PHA-E and PHA-EZnO films, Φ6 lysate was incubated with the films (each active film separately) according to the ISO 22196-2011 standard [34], while Φ6 particles were incubated with a PHA film at the same time. The LB broth was introduced into BioSan bioreactors (BS-010160-A04, BioSan, Riga, Latvia). Then, the *P. syringae*/host overnight culture was added to 10 mL of LB broth and incubated at 28° until OD (optical density) = 0.2. In total, 4 Φ6 lysates were amplified in the *P. syringae* culture (1 lysate—after its incubation with the PHA sample; 1 lysate—after its incubation with the PHA-E sample; 1 lysate—after incubation with the PHA-ZnO sample; 1 lysate—after its incubation with the PHA-EZnO sample). As the next step of the investigation, 10 µL of Φ6 phage lysate (MOI = 1) was added to the bacterial culture (OD = 0.2) and incubated for 24 h at 28 °C.

## 3. Results

### 3.1. Results of Studies on the Active Additives

The additives were characterized (FTIR, thermal properties) as well as their antimicrobial activity, which was investigated before their introduction into the polymer matrix. The main goal of this preliminary task was to investigate themutual interactions between the herb extracts and ZnO. The preliminary microbial analysis of the extract-based additives is presented in more detail in the Supplementary Materials.

#### 3.1.1. FTIR-ATR of the Additives

The FTIR-ATR spectra of Atmer, the mixture of the dried extracts and the mixture with ZnO is presented in the Figure S3. There were no significant differences between the mixture and mixture/ZnO except a slight shift in peak for the herbal mixture at 1057 to 1044 cm^−1^, for the mixture with the nanofiller. This peak is assigned to the stretching vibration of the bonding between carbon and hydrogen groups and -C-O groups from the ether and ester groups, e.g., from tannins, flavonoids [35] and chlorophylls. The shift indicates some interaction between the extract components and ZnO nanoparticles.

**Figure 1:**
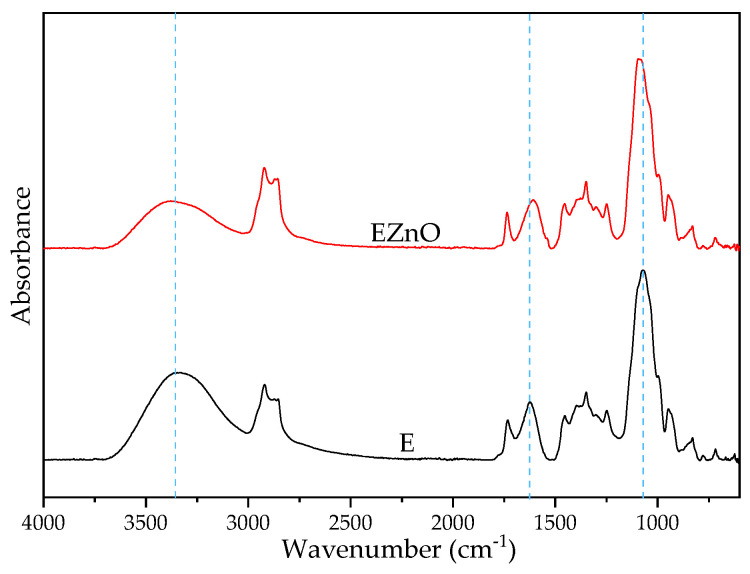
FTIR-ATR spectra of the additives: **E**—herbal extract mixture (in Atmer), **EZnO**—extract mixture/ZnO system (in Atmer).

In Figure 1, it can be seen that there are some band shifts from 3343, 1732, 1642 and 1092 cm^−1^ for E to 3389, 1738, 1609 and 1072 cm^−1^ for EZnO, respectively. The shift of the broad band assigned to OH groups (3300–3000 cm^−1^) toward a higher wavenumber for the sample with the ZnO presence may indicate some H-bond weakening in the extract in the Atmer by the ZnO particles. It may also be related to the high dispersion degree of ZnO in this additive system, due to the presence of the compounds from the extracts [36]. Moreover, the peak intensity was lower, indicating the participation of a new bonding formation after nanofiller addition. The band assigned to the carbonyl groups was shifted into lower wavenumbers, which may be a result of the formation of a complex between the extract compounds and zinc oxide.

#### 3.1.2. TGA Results

**Figure 2:**
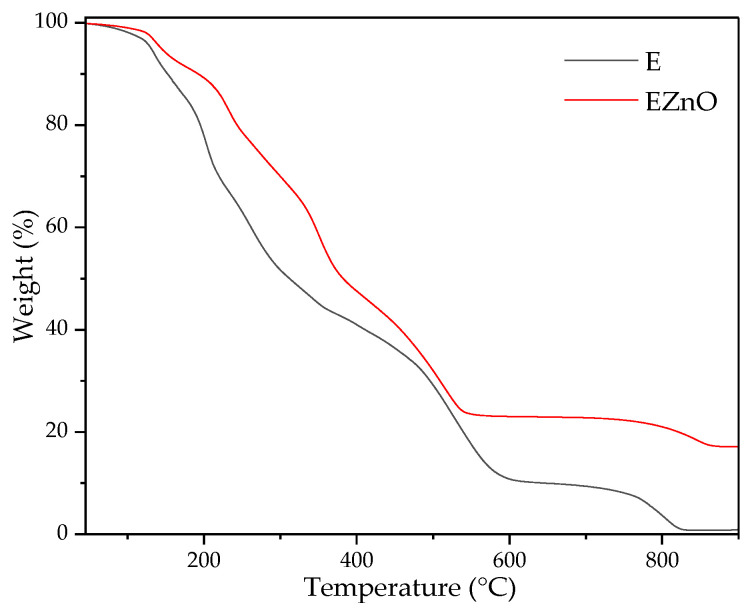
TGA curves for the modifiers: **E** (herbal extract + Atmer) and **EZnO** (herbal extract + Atmer + ZnO nanoparticles).

The formation of the complex between ZnO and the organic mixture was confirmed by the TGA results. It can be seen from Figure 2 that EZnO is much more thermally stable than E. This phenomenon is especially necessary when the additives are processed at higher temperatures, in this case, in the extrusion with PHB. Similar results were obtained in Ref. [36], where the olive leaf extract was much more thermally stable after 3 wt % of ZnO addition.

### 3.2. Results of Film Characterization

The obtained films were quite smooth, matt and a little opaque. PHA and PHA-ZnO had a light ecru color and PHA-E and PHA-EZnO had an intense green color, which changed into light olive green after a few days of storage without any covering. What is interesting is that the PHA-EZnO sample was highly transparent and not as opaque as the rest of the samples.

#### 3.2.1. Mechanical Tests

Table 2 presents the results of the tensile test of the films obtained via cast extrusion.

The content of the additives in the matrix is 0.04 pph of ZnO (+1.33 pph of Atmer) and ca. 1 pph of E and EZnO. The modification did not influence the film’s mechanical properties significantly, except for the hybrid system EZnO. The presence of this additive in PHB significantly increased its tensile strength (TS) (24 MPa). The increase in YM and TS without decreasing the elongation at break (EB) (which, generally, leads to higher toughness) may have been caused by some complex formation between the extracts and ZnO particles that affected not only the higher thermal stability of EZnO (Figure 2) but also facilitated additive distribution in the polymer matrix (see the SEM results below). As can be seen in Figure S4 in the Supplementary Materials, the herbal mixture prevents the agglomeration formation of ZnO particles. This increase in mechanical properties may also indicate an anti-plasticization effect resulting from strong intermolecular attractions and the reduction in free volumes at low concentrations of the additives, which are below the threshold condition for plasticization [37]. In the case of ZnO, only a higher content affected the mechanical properties. In the work of Berrabah et al. [38], the addition of less than 1.5 pph of ZnO improved the mechanical properties of poly(3-hydroxybutyrate-co-3-hydroxyhexanoate), while higher amounts led to the material’s brittleness. The improvement of the mechanical properties of PHA composites obtained via solventless methods was also obtained in Ref. [35], where the highest improvement was obtained for 0.4% of micro-sized hexagonal ZnO crystals in the polyester composite. It is worth highlighting that the addition of the extract mixture did not affect the mechanical properties as in other works, e.g., rice extracts added to PLA [39] and phenolic compounds added to PHBV [21] worsened the mechanical properties of the studied materials.

#### 3.2.2. SEM Results

**Figure 3:**
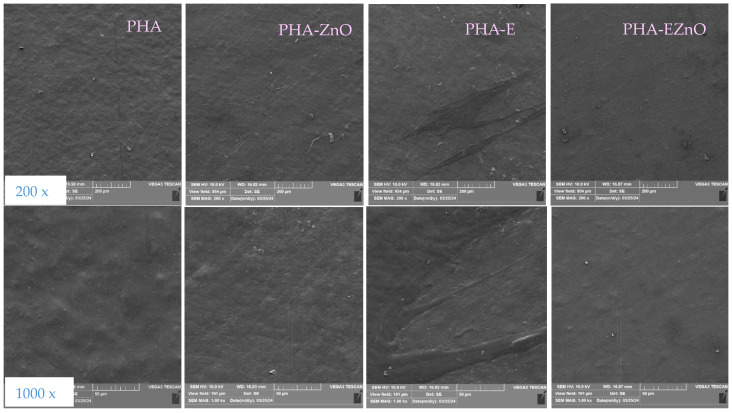
SEM micrographs for the films at 200× magnitude (first row) and 1000× magnitude (second row).

SEM analysis was carried out to visualize the surface of unmodified PHA films and PHA with the incorporated active agents (Figure 3). It can be observed that the PHA film exhibited a smooth morphology. Similar results were also noted by Madbouly et al. [40]. Conversely, Shamala et al. [41] indicated that polyhydroxyalkanoates exhibited a surface with high porosity. The PHA-ZnO films were less smooth but still homogenous, due to a good distribution of the filler during the extrusion process. Additionally, aggregates of small, white dots were visible on the biopolymer surface. It was assumed that the partial agglomeration of zinc oxide nanoparticles can develop in the matrix. The presence of zinc oxide nanoparticles in polymer films was confirmed by Mania et al. [42], who mentioned that ZnO nanoparticles were visible in the form of white dots on a gray background (of the film). The Authors observed greater amount of nanoparticles as white-clouded dots with a non-uniform distribution, indicating the partial agglomeration of the filler. No agglomeration was observed in another work describing PLA/ZnO composites [43]. The surface of the PHA-E films was less homogenous and smooth, which confirms the poorer miscibility of the herbal extract mixture in the matrix. In comparison with PHA-E, the surface of the PHA-EZnO was much smoother and almost uniform, without the agglomeration of zinc oxide nanoparticles and extract phases in the matrix. This may be related to the hybridization of the extract mixture with ZnO, which consequently resulted in better miscibility [36] of the additive in the PHA matrix. The good modifier distribution may be related to the higher tensile strength of PHA-ZnO among the samples (Table 1).

#### 3.2.3. Thermal Characterization

**Figure 4:**
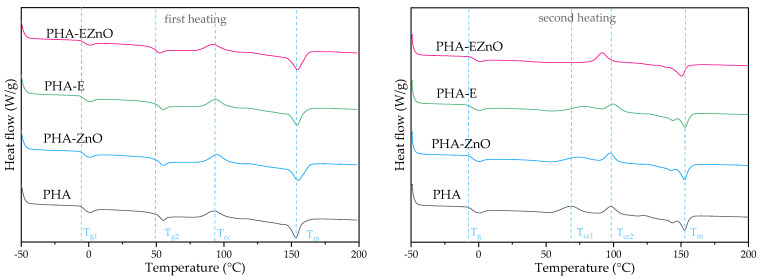
DSC diffractograms for the films (first and second heating runs).

Calorimetric diffractograms of the films are presented in Figure 4. The phase transitions were dominated by the PLA fraction [6,44]. Two glass transition peaks were observed for the first heating, indicating that the polyesters were non-miscible in the bioresin (Ecomann EM 40000). A value for T_g1_ at ca. −7 °C was assigned to the PHA fraction and T_g2_ at ca. 50 °C to the PLA fraction, and there were similar T_g_ values for all samples. Comparing the native polyester film with the modified analogs, there was a small shift of cold crystallization of the PLA fraction peak [6,45] from 93.0 °C for PHA to 95.5, 94.3 and 92.4 for PHA-ZnO, PHA-E and PHA-EZnO, respectively. It may be caused by the presence of the additive in the polymer matrix; this slightly affected the crystallization, but the shift in the temperature was also barely visible, which may have been caused by the low content of the additive. The melting points (T_m_) of the PHA films were not affected by the addition of zinc oxide or the mixture of herbal extracts in the Atmer carrier. Only PHA-ZnO exhibited a slightly higher T_m_, which might have been caused by the formation of more complete crystals [46]. As can be seen in Figure 4, in the second heating, only the glass transition of PHA is visible, which is the dominant fraction, but the second cold crystallization peak (T_cc1_) and two overlapped peaks T_m_ have also appeared. The T_cc1_ peaks for PHA-ZnO and PHA-E are “more flat”, which may indicate a less compact structure, caused by the additive’s presence [47]. Similar results were obtained in Ref. [6]. The authors explained that this small peak was assigned to less stable crystals. What is interesting is that in this study, there was only one T_cc_ and a single T_m_ peak for PHA-EZnO, and these peaks were shifted to lower temperatures in comparison with other materials. These changes may be the result of the less heterogenous character of the blend (e.g., better miscibility of the polyester fractions), and these findings somehow correspond with the TGA results. However, this phenomenon needs further, deeper studies.

Table 3 shows the results of thermo-oxidative stability determination and OIT values, depending on the additive type. The parameters for the sample that was modified with the extract mixture exhibited slightly lower stability than PHA, whereas films with the addition of zinc oxide exhibited higher stability. The sample most resistant to thermo-oxidation was PHA-EZnO. In the case of extract-based additives, the results are opposite to those for films obtained in Ref. [48], where PHB modified with grape seed extract exhibited a higher OIT than the pure sample (although the amount of the extract was much higher). In the case of ZnO, their type and modification affect the OIT of the composites. Yao et al. [49] reported that hexagonal column-shaped ZnO particles affected the OIT, whereas tetrapod ZnO whiskers did not influence these parameters. In Ref. [50], ZnO, combined with the stabilizer Hostavin N321, increased the OIT of an ethylene-norbornene copolymer more significantly than pure ZnO.

**Figure 5:**
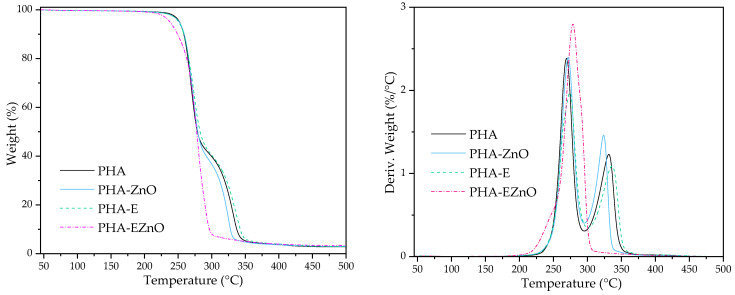
TGA (**left**) and DTG (**right**) of the films (heated in an air atmosphere).

A TGA was performed to examine the thermal stability of the films. This is particularly important from the processing point of view, especially when natural-based extracts that are rich in active compounds are used. The thermograms are presented in Figure 5. PHA films, except for PHA-EZnO, are thermally stable up to 240 °C. Their thermal decomposition temperature is much higher than their processing profile, so there is no risk of the occurrence of material degradation during their reprocessing, e.g., for packaging (thermoforming or sealing). One of the samples, PHA-EZnO, started to decompose at ca. 230 °C. The beginning of this process was more pronounced, as shown in the DTG curve. The peak for PHA with the hybrid additive started increasing at ca. 220 °C, whereas the increase for the rest of the samples began at 232 °C.

Moreover, for PHA-EZnO, there was only one step of decomposition (as well as one peak on DTG), whereas the other samples exhibited two peaks; the second one is at ca. 286 °C, with ca. 40% of the sample’s weight (SI, Table S3). The first weight loss is related to the decomposition of PHB, and the second one came from the PLA’s degradation [42]. The disappearance of the two peaks and the appearance of one peak for PHA-EZnO is all the more interesting. Our assumption is based on the influence of ZnO on thermal degradation. Comparing PHA with PHA-ZnO, it can be seen that for the first peak from the PHA fraction, the additives did not influence its thermal stability, whereas, for the PLA fraction, the maximum of the second peak was at ca. 332 °C for PHA and ca. 325 °C for the analog with the nanofiller. This phenomenon was described by Anžlovar et al. [51]. The authors evidenced the degradative influence of ZnO addition on PLA, while PHBV was much less sensitive. Additionally, in Ref. [43], the authors demonstrated the dramatically lower thermal stability of PLA films after introducing ZnO. The combined additive, EZnO, due to the high dispersion degree in the polymer matrix (see the SEM and mechanical test results), can increase the interphase between the additive and the polymer, facilitating the faster degradation of PLA. Moreover, this finding may somehow be related to the DSC results, where the PHA-EZnO curves indicated better miscibility than could probably have been caused by partially degraded PLA chains, as well as T_cc_ and T_m_ having shifted toward lower temperatures (2nd heating).

#### 3.2.4. Barrier Properties

Table 4 presents the results of the studies of barrier properties regarding oxygen (OTR) and moisture (WVTR). These features are important from a packaging point of view. Oxygen can cause organoleptic changes (e.g., lipid oxidation or loss of aroma) and moisture, facilitating food spoilage. The OTR is in the range of 57–70 cm^3^/m^2^/24 h and the values are much lower than for polyolefins [52] and lower than PLA films [6]. The obtained results are closer to the values for aromatic polyesters like poly(ethylene terephthalate) (PET) [53]. In general, blending PLA with PHA (PHB) led to the improvement of barrier properties toward oxygen and moisture, compared to pure PLA [6]. Comparing the modified films, it can be seen that the additives with zinc oxide slightly decreased the OTR, and the highest improvement was obtained for PHA-EZnO. A similar trend can also be observed in the case of WVTR. This may be related to the high degree of additive distribution in the polymer matrix and the improved interfacial adhesion and free volume reduction in the composite [37]. These results correspond with the mechanical test results and SEM. Dai et al. reported that the introduction of zinc oxide led to a decrease in moisture permeability, but the addition of an extract from pomegranate peels increased this parameter [43].

#### 3.2.5. Optical Properties 

**Figure 6:**
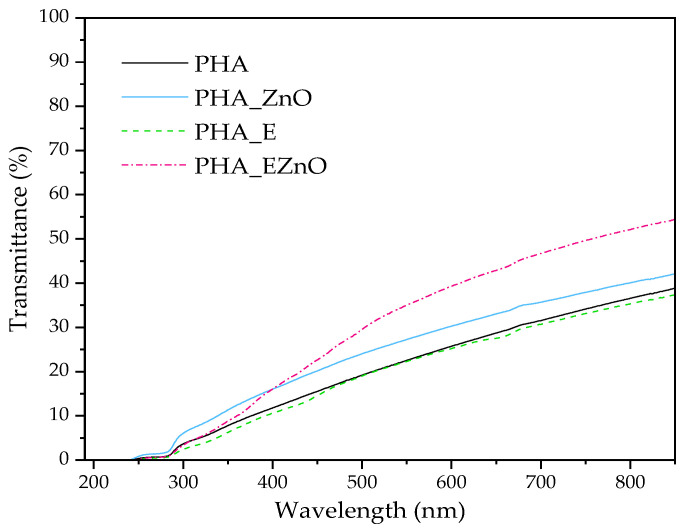
UV-Vis spectra of the films.

The UV-Vis spectroscopy (transmittance) results are presented in Figure 6. It can be seen that all studied samples exhibited barrier properties toward UV radiation. This property can be an advantage, e.g., in food or cosmetic packaging, especially for products that are sensitive to UV aging, which can cause the loss of active ingredients or organoleptic changes. Comparing the transmittance in visible light, it can be seen that the opaque, matt appearance of the films PHA, PHA-ZnO and PHA-E is reflected by their low transparency (at 700 nm) values, in the range of ca. 31–36% (Table 5).

Analyzing the additive effect, it can be seen that samples with zinc oxide exhibited higher transparency (T). The highest parameter value (47%) was observed for the PHA-EZnO film. This could be related to the high degree of additive distribution in the polyester matrix (see the SEM results) and the changes in the miscibility of the polyester fractions in the matrix (see DSC, TGA and SEM). Similar results, where the transparency of the PLA/PHB blend was a bit higher after the additive introduction, were obtained in Ref. [6], where the blend was mixed with 15% of limonene.

Due to the addition of an intensively colored modifier originating from natural resources, the color of the native PHA material was altered after functionalization with the extract-based systems. The original color of PHA is visible as a creamy tint and, on the CIELab scale, is reported as a b* close to 2. At first, directly after PHA processing (regranulation), the samples with the extracts were green, due to the color of the modifier. The green color for PHA-ZnO was much brighter and more intense and for PHA-E, it was slightly olive-green (a lower b* value assigned to yellow and a* shifted toward the lower minus value assigned to green). However, after a few days of storage, the color of the films changed to a more brownish color (with lower b* values and a* shifted toward red). It can be seen that after some time, the colorification intensity was less intense (lower ∆E, YI and C* values). This phenomenon may be related to the formation of the chlorophyll derivatives pheophytin or pheophorbide in herbal extract-based systems [54,55].

### 3.3. Antimicrobial Activity of the PHA Films with Active Agents in the Biopolymer Matrix

**Figure 7:**
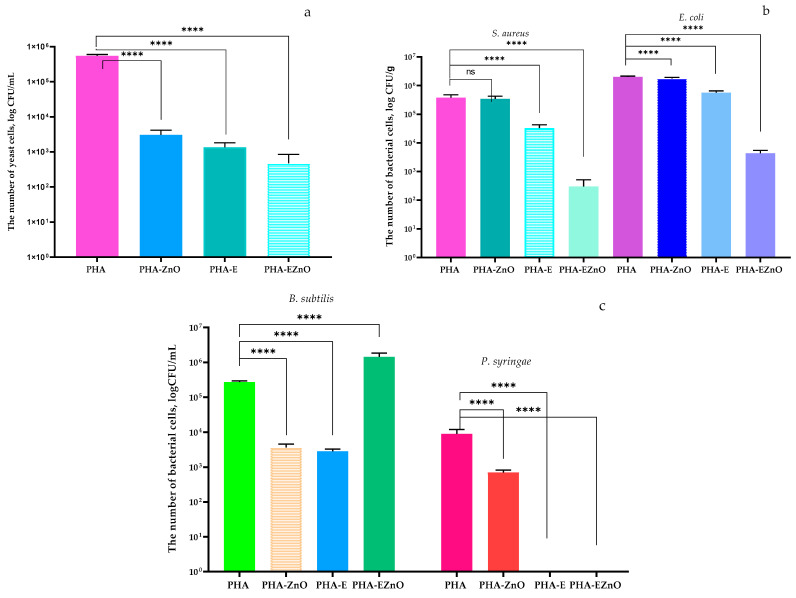
The influence of the films on: (**a**) *C. albicans*; (**b**) *S. aureus* and *E. coli*; (**c**) *B. subtilis* and *P. syringae*. PHA—PHA film; PHA ZnO—PHA film with ZnO in the biopolymer matrix; PHA E—PHA film with a mixture of *Hypericum* L., *Chelidonium* L. and *Urtica* L. extracts; PHA EZnO—PHA film with a mixture of extracts with ZnO. One-way ANOVA; ****—*p* < −0.0001; not significant (ns)—*p* > 0.5.

A microbiological examination of the films determined that the PHA-ZnO film was effective against the *C. albicans* strain because it reduced the number of yeast cells when compared to PHA films (control samples) (Figure 7a). The statistical analysis confirmed that the differences between the numbers of the microorganism cells were significant. Similar results were observed in a previous study [56], which demonstrated that a composite PLA film with zinc oxide decreased the number of *C. albicans* cells significantly. The PHA film with the addition of the *Hypericum* L., *Chelidonium* L. and *Urtica* L. extract mixture was found to be more active against *C. albicans* than the PHA-ZnO film. Moreover, PHA-EZnO was the most effective sample against yeast cells, confirming the cooperative effect of these two active agents. As reported by Aldalbahi et al. [57], Cs/PVP films containing *E. citriodora* leaf extracts were active against *C. albicans*.

The microbiological analysis also indicated that PHA films containing ZnO nanoparticles were not effective against the *S. aureus* strain because they did not inhibit the growth or even reduce the number of bacterial cells (Figure 7b). Similar results were noted when analyzing the activity of the films against *E. coli*. A previous study [56] revealed that PLA films with nano-ZnO reduced the number of cells of *S. aureus* (2 log) and *E. coli* (decrease lower than 2 log). However, the amount of nano ZnO in the biopolymer matrix was higher (0.14 pph) than that introduced into the PHA matrix in the current study (0.04 pph). Similarly, Mania et al. [41] noticed that PE with zinc oxide (1.5% and 3%) caused a slight (0.28 log and 1.16 log) and low (2.1 log and 2.73 log) decrease in the number of *S. aureus* and *E. coli*, respectively. It should be mentioned that the authors found *E. coli* to be more sensitive to nanofiller than *S. aureus*. To summarize, the PHA films were not effective against the mentioned microorganisms. As Mania [39] reported, the global migration of zinc oxide nanoparticles from the polymer matrix (PE) was 0.155 (to water) and 0.0017 mg/dm^2^ (to 95% ethanol). The PHA films with the addition of the extract mixture were found to be active against the *S. aureus* and *E. coli* strains. As is shown in Figure 7b, the number of microorganisms was significantly decreased. The PHA films containing both active agents, ZnO nanoparticles and herbal extracts, were confirmed to be effective films against *S. aureus* cells. Although these active films did not inhibit the growth of Gram-positive bacteria completely, they significantly reduced their number. Similar results were noted when the activity of the films against *E. coli* was analyzed. However, the PHA-EZnO films were more effective against *S. aureus* than against *E. coli*. A previous study [26] demonstrated that a PE film modified with a mixture of scCO_2_ extracts (from raspberry seeds, pomegranate seeds and rosemary) with confirmed synergistic effects [58] inhibited the growth of *S. aureus* and reduced the number of *E. coli* cells. As is emphasized in Figure 7b, the PHA-EZnO films were more active than the PHA-E films against Gram-negative cells. These observations confirmed the synergistic effect of plant extracts and zinc oxide nanoparticles as active agents. Similar results were observed in a previous work [19], where nano-ZnO increased the activity of geraniol and carvacrol against the *E. coli* and *S. aureus* strains. Dai et al. [43] observed that PLA with nano-ZnO greatly decreased the growth rate of *S. aureus*, indicating that the PLA/ZnONPs film had significant antibacterial effectiveness against Gram-positive bacteria. However, this antibacterial activity weakened with time. The authors observed that the addition of pomegranate peel extract (PPE) into PLA made it active against *S. aureus.* As the authors assumed, this might have been related to the gradual release of PPE from the matrix. Additionally, they suggested that the polyphenol compounds from PPE exerted antibacterial activity. It should be underlined that the antibacterial activity of the composite film was greatly enhanced by adding ZnONPs into the PLA with the PPE extract. Their results also confirmed the cooperative effect between plant extracts and zinc oxide nanoparticles in the polymer matrix. However, contrary to the results of the current study, the authors’ findings demonstrated that active PLA films were more effective against *E. coli* than *S. aureus*.

The analysis of the antimicrobial properties of the films against *P. syringae* demonstrated that a PHA film containing zinc oxide nanoparticles was effective against the *P. syringae* strain because it significantly reduced the number of bacterial cells (Figure 7c). The PHA film with the addition of the extract mixture was even more active against *P. syringae*. As can be seen in Figure 7c, it inhibited the growth of microorganisms completely. Moreover, the PHA film that was functionalized with the hybrid system of both active agents was also significantly effective against *P. syringae* cells, inhibiting their growth. The opposite results were observed when an analysis of the activity of the films against *B. subtilis* was performed. As is emphasized by Figure 7c, the PHA-EZnO film was not effective against Gram-positive cells. It did not decrease the bacilli cells even slightly. On the contrary, the number of microorganisms was observed to be increasing, meaning that the film stimulated bacterial growth. Heydari-Majd et al. [59] prepared PLA films with the addition of 1.5% *w*/*w* zinc oxide nanoparticles and varying concentrations (0.5, 1.0 and 1.5% *w*/*w*) of *Zataria multiflora* Boiss essential oil and *Menthe piperita* L. essential oil. The authors’ results confirmed a cooperative effect between the active agents, due to increased activity against *B. cereus* and *P. aeruginosa*. Contrary to the results reported in the current study, Gram-negative *P. aeruginosa* was more resistant to the films than Gram-positive *B. cereus*. The authors suggested that this might have been attributable to the presence of an additional external membrane surrounding the cell wall in Gram-negative bacteria, which may restrict the diffusion of hydrophobic substances across the lipopolysaccharide layer. Moreover, the PHA-ZnO and PHA-E films were confirmed to be active against *B. subtilis*. Both active films decreased the number of these bacteria significantly, which was confirmed by the statistical analysis. CO_2_ extract-modified PE film was effective against both *B. subtilis* and *P. syringae* [26]. However, the material obtained in this previous work was less effective than the PHA-E films. On the other hand, similar results were observed by Aldalbahi et al. [57], who reported that biodegradable films made of chitosan and polyvinylpyrrolidone matrices, with the addition of *E. citriodora* leaf extracts, exhibited effectiveness against *P. aeruginosa* and *B. subtilis*.

**Figure 8:**
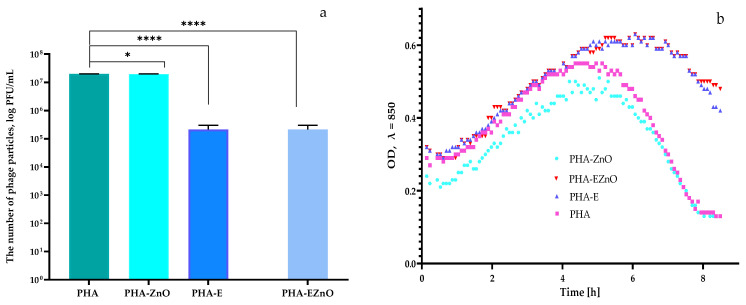
(**a**) The influence of the films on the Φ6 phage (Φ6). One-way ANOVA; ****—*p* < −0.0001; *—*p* < 0.1); (**b**) The optical density (OD) over time of *P. syringe* with Φ6 phage after its incubation with unmodified and modified films. PHA—PHA film; PHA-ZnO—PHA film with ZnO in the biopolymer matrix; PHA-E—PHA film with a mixture of extracts; PHA-EZnO—PHA film with a mixture of extracts and ZnO nanoparticles.

Our analysis of the antiviral properties of active films indicated that the PHA-ZnO film was not effective against phi6 lysate because it did not inactivate the bacterial virus particles or did not even reduce the titer (Figure 8a). However, statistical analysis demonstrated that the differences between the active viral particles were significant. The PHA-E and PHA-EZnO films were noted to be active against the Φ6 phage, due to the decreased titer of phi6 lysate (Figure 8a). Similar results were found in the previous study [26], which showed that the number of bacteriophage particles decreased after their incubation with the functionalized film when compared to the number of particles, which was noted after their incubation with the non-active PE film.

The results of the antiviral analysis demonstrated that an OD fall was observed after 5 h of incubation of *P. syringae* with the phages, which were incubated with the control sample—with the PHA or PHA-ZnO samples confirming that these two films were not effective against viral particles (Figure 8b). Furthermore, the titers of the phage lysate observed with the PHA and PHA-ZnO films were only slightly different (Figure 8a). In the case of the PHA-E and PHA-EZnO films, an OD fall was observed after 7 h of incubation of *P. syringae* with the phi6 bacteriophages that were incubated with the above-mentioned active films (Figure 8b). Additionally, the titers of the Φ6 lysate observed for the PHA-E and PHA-EZnO samples significantly decreased when compared to the control sample. These results confirmed that films containing plant extracts or plant extracts and zinc oxide nanoparticles were active against a bacterial virus. Contrary results were reported in a previous study [26], which showed an OD fall for the *P. syringae* culture with the addition of Φ6 lysate, which was incubated with the active PE film. Comparing the current results with the previous findings, it should be underlined that functionalized PE films had higher antiviral properties than PHA-EZnO. Unfortunately, the current work also showed that a cooperative effect between the active agents (plant extracts and ZnO nanoparticles) was not noted.

The PHA-E and PHA-EZnO showed moderate effectiveness against the Φ6 bacteriophage, which is enveloped by a lipid, external layer and may be considered as a SARS-CoV-2 surrogate [60,61]. Based on these results, it may be assumed that PHA films that were effective toward Φ6 particles could also lead to a decrease in the number of SARS-CoV-2 particles. These active PHA films could limit the spread of SARS-CoV-2 when new variants appear or when there is a risk of future epidemic or pandemic outbreaks [60,61,62].

## 4. Conclusions

A bioresin based on poly(hydroxybutyrate)–P(3HB-*co*-4HB) with ca. 40% of PLA was effectively functionalized with a hybrid system formed by the herbal mixture of *Hypericum* L., *Urtica* L. and *Chelidonium* L., along with a ZnO nanofiller. Three herb extracts were selected to broaden their antimicrobial activity spectrum. They were introduced to the Atmer carrier (a commercial additive for plastics) to obtain the E modifier or were mixed with a nano-ZnO, forming the EZnO modifier. The E and EZnO were analyzed with FTIR and TGA, and the results of the investigation revealed interactions between the extracts and the nanofiller, leading to the higher thermal stability of the hybrid system. The additives, especially EZnO, were well dispersed in the polymer matrix (SEM) and did not affect the film morphology (via FTIR). The hybrid modifier influenced the mechanical, barrier and optical properties. PHA-EZnO exhibited the highest tensile strength and the lowest OTR and WVTR values, as well as the highest transparency. What is interesting is that the addition of EZnO significantly affected thermal stability and there was only one peak of decomposition, whereas, for the rest of the samples, there were two peaks assigned to the polyester fractions. Similar results were obtained from the DSC, where the T_m_ for PHA-EZnO stands out among the rest of the samples.

The microbiological examination indicated that these modified films exhibited antimicrobial activity, depending on the additive used, although the functionality was different toward the studied microorganisms. PHA-E inhibited the growth of *P. syringae* cells and decreased the number of *S. aureus*, *E. coli*, *B. subtilis* and *C. albicans* strains, while PHA-EZnO inhibited not only the mentioned strains (except of *B. subtilis*) but also the *P. syringae* cells, indicating high levels of activity against this strain. In the case of *S. aureus*, *E. coli* and *C. albicans*, it should be underlined that the antimicrobial activity of the films with the combined additive (EZnO) was higher than the effectiveness of the films where the additive components of zinc oxide and the herbal mixture were introduced separately. The findings confirmed the synergistic effect between the components. Antiviral tests showed that PHA films modified with both extract-based additives did not eliminate the phi6 bacteriophage particles completely, but they did decrease their number, confirming moderate antiviral effectiveness.

## Figures and Tables

**Table 1:** The amounts of the components of the polyester blend (pph).

Components	ZnO	E	EZnO
Atmer 110	1.33	1.33	1.33
Extract mixture *	-	1	1
ZnO	0.04	-	0.04

* Dried residue after total ethanol evaporation.

**Table 2:** Tensile test results (with standard deviation values in the brackets).

Sample Acronym	Young’s Modulus (MPa)	Tensile Strength (MPa)	Elongation at Break (%)	Thickness (µm)
PHA	846 (±79.7) ^a^	20.6 (±1.39) ^b^	4.2 (±0.28) ^a^	157
PHA-ZnO	965 (±98.1) ^a^	20.6 (±2.18) ^b^	5.2 (±1.18) ^a^	130
PHA-E	939 (±52.9) ^a^	20.9 (±1.23) ^b^	4.6 (±0.57) ^a^	156
PHA-EZnO	982 (±74.1) ^a^	24.3 (±2.08) ^a^	4.7 (±0.16) ^a^	150

a–b—Averages marked with the same letters do not differ significantly from each other for *p* < 0.05.

**Table 3:** Oxidation peak parameters (isothermal OIT).

Sample	Time to Oxidation Peak (min)	End Set (min)
PHA	2.62	13.4
PHA-ZnO	3.36	15.7
PHA-E	2.53	12.9
PHA-EZnO	6.5	14.8

**Table 4:** Oxygen transmission rate (OTR) and water vapor transmission rate (WVTR) for the films (standard deviation values are shown in the brackets).

Sample Acronym	OTR _RH 0%_ (cm^3^/m^2^/24 h)	WVTR _RH 100%_ (g/m^2^/24 h)	WVTR _RH 90%_ * (g/m^2^/24 h)
PHA	70.7 (±7.97)	55.9 (±0.84)	50.3 (±0.78)
PHA-ZnO	60.7 (±3.81)	55.9 (±0.85)	51.3 (±2.53)
PHA-E	72.7 (±9.15)	53.6 (±1.23)	48.2 (±0.13)
PHA-EZnO	57.4 (±0.69)	49.4 (±4.60)	44.4 (±4.10)

* Compensated.

**Table 5:** Results of color measurement on the CIELab scale and the transparency (T—transmittance at 700 nm) of the films (standard deviation values are shown in the brackets).

Sample Acronym	L*	a*	b*	C*	YI	∆E	T _(700 nm)_
PHA	98.2 (±0.06)	0.17 (±0.02)	1.92 (±0.20)	1.95	2.82	-	32%
PHA-ZnO	97.8 (±0.15)	0.10 (±0.04)	1.50 (±0.26)	1.50	2.19	0.44	36%
PHA-E	94.6 (±0.28)	−5.70 (±0.35)	16.32 (±0.93)	17.29	24.64	15.86	31%
PHA-E stored	94.8 (±0.23)	−2.87 (±0.30)	13.33 (±1.28)	13.65	20.09	12.16	-
PHA-EZnO	92.1 (±0.03)	−7.40 (±0.38)	21.43 (±1.13)	22.60	33.24	21.67	47%
PHA-EZnO stored	92.1 (±0.15)	−1.92 (±0.30)	14.72 (±1.09)	14.84	22.83	14.15	-

## Data Availability

Data are contained within the article and Supplementary Materials.

## Supplementary Materials

The following supporting information can be downloaded at: https://www.mdpi.com/article/10.3390/polym16141954/s1, Figure S1: The growth of *S. aureus* in real-time in the presence of the mixture of *Hypericum* L. (HE), *Chelidonium* L. (ChE) and *Urtica* L. (UE) extracts, C-control; Figure S2: The growth of *E. coli* in real-time in the presence of mixture of *Hypericum* L. (HE), *Chelidonium* L. (ChE) and *Urtica* L. (UE) extracts, C-control; Figure S3. FTIR of additives (E, EZnO) and their individual components; Figure S4: SEM micrographs of of ZnO (left), dried mixture of extracts (middle) and the mixture with ZnO (right); Figure S5: FITR-ATR spectra of PHA-based films; Table S1: DSC results for the first and second run; Table S2: MFI values for native granulate and regranulates; Table S3: TGA results.
