# Supplementary material for: Cast Extruded Films Based on Polyhydroxyalkanoate/Poly(lactic acid) Blend with Herbal Extracts Hybridized with Zinc Oxide
Source: Polymers (Basel). 2024 Jul 9;16(14):1954. doi: 10.3390/polym16141954 (PMC11281330; doi:10.3390/polym16141954)
Supplement: Supplementary file 1 [file polymers-16-01954-s001.zip › polymers-3044054-supplementary.pdf]

SUPPLEMENTARY INFORMATION

Cast extruded films based on polyhydroxyalkanoate/poly(lactic acid) blend with herbal extracts hybridized with zinc oxide

Magdalena Zdanowicz [1]*, Małgorzata Mizielińska[1] and Agnieszka Kowalczyk [2]

METHODS

SI.1. The extracts preparation

The plant extracts were prepared using dry plants: *Hypericum* L., *Urtica* L. and *Chelidonium* L. The amount of 150 g of dry herbs was introduced separately into 900 mL of 98% ethanol. Then, the ethanolic systems (in sealed bottles) were introduced into a Microwave (Amica, Wronki, Poland) for 15 min at 70 °C. As a continuation of extraction process, the bottles were placed in a shaker (Ika, Staufen im Breisgau, Germany) for 1 hour at 70 °C (150 rpm). The extraction/process was accomplished according to the procedures described by the authors [17-18,25] with a slight modification. After extraction, the solid residue of the herbs was separated from the extracts using a Büchner funnel. Then, 300 mL of each extract was mixed together to obtain 900 mL of the extracts' mixture (300 mL of the mixture was used to the preliminary microbiological analysis; 600 mL of the mixture was left to the experiments to the current study). In the next step, solvent in 300 mL of the mixture was evaporated. Simultaneously, 300 mL of each extract (separately) were left to evaporate the ethanol. Then, 10 wt% solution of each extract (separately) in DMSO and 10 wt% solution of the herbal extracts' mixture in DMSO (to maintain the liquid state of extracts as well as to avoid adhesion to the glass) were prepared to analyze the antimicrobial effectiveness of pure additives before their introduction into the bioresin. 300 mL of the ethanolic mixture (which was not dried), was left for the next experiments.

SI.2. The preliminary antibacterial activity analysis

The preliminary antibacterial properties analysis of pure herbal extracts (separately) and their mixture was performed using BioSan bioreactors (BS-010160-A04, BioSan, Riga, Latvia). As a first step of the experiment, 1 g of 10 wt% of each plant extract (separately) in DMSO was introduced into the 9 g of LB medium (to obtain 1% solutions). As a second step of the experiment, 1 g of 10 wt% solution of plant extracts mixture in DMSO was introduced into the 9 g of LB medium (to obtain 1% solution). Simultaneously, 1 g of DMSO was introduced into the 9 g of LB medium. It was important to investigate if DMSO influences on the growth of bacterial cells. As the next step, overnight cultures of *S. aureus* and *E. coli* ($1.2\text{x}10^8$ CFU/mL, separately) were prepared. Then, 100 µL of each culture (separately) was added to LB medium (control sample), LB medium with DMSO and 1% of the herb extracts mixture in LB medium (to obtain inoculum of $1.2\text{x}10^6$ CFU/mL). The test tubes with analyzed samples were introduced into the BioSan bioreactors and left for 24 h incubation at 37 °C.

RESULTS

SI.3. The preliminary antibacterial activity analysis

The preliminary microbial analysis was performed using two bacterial cells, *S. aureus* as a member of Gram-positive bacteria and *E. coli* as a member of Gram-negative microorganisms. Previous works confirmed that there was a synergistic effect between plant extracts (ethanolic or CO_2) when they were introduced into the polymer matrix [26] or the active coatings [18,24]. Based on these results, it was assumed that a synergistic effect between extracts in the mixture (which was supposed to be used as an active agent in the next experiments) of *Hypericum* L., *Chelidonium* L. and *Urtica* L. would be confirmed. Simultaneously, the antimicrobial activity of each extract (separately) was analyzed. After ethanol evaporation, the extracts mixture and/or each extract (separately) was dissolved in DMSO, and the 10% solution of the extract/extracts' mixture was used to analyze their antibacterial effectiveness (to obtain the final concentration of the extract/extracts' mixture in LB =1%). It was also assumed that 1% solution of DMSO should be examined to find out if DMSO was active against bacteria. This is why experiments were performed simultaneously (LB medium with DMSO containing each, neat extract, extracts mixture and LB with DMSO).

The results of the preliminary, antimicrobial analysis demonstrated that the mixture of *Hypericum* L., *Chelidonium* L., *Urtica* L. extracts and the neat extract of the *Chelidonium L.* were active against *S. aureus* strain. As was seen in Figure S1, the fast growth of *S. aureus* in LB medium was observed. However, the microorganisms grew lower in LB with DMSO meaning that DMSO as a solvent reduced the initial number of bacterial cells. Analyzing OD over time of Gram-positive cells in LB medium with DMSO and the herbal extracts mixture, it was noted that this parameter decreased. Initial OD value of *S. aureus* culture with extract mixture/neat extracts was high due to the herbal mixture color (dark green). It was not caused by the number of microorganisms cells. As was shown in Figure 1, OD did not increase over time confirming that the bacteria were not active after their incubation with extract mixture and with neat *Chelidonium L.* extract. The results clearly confirmed that *Chelidonium* L. extract and th extracts mixtures inhibited the growth of *S. aureus*. Analyzing the influence of the *Urtica* L. neat extract on the *S. aureus strain*, the fast growth of microorganisms was observed until 2 hours of incubation. OD fall was noted only after 2.5 hours of cultivation. Similar results were noticed for *Hypericum* L., extract. However, the initial growth of microorganisms incubated with this extract was lower than bacteria cultivated with *Urtica* L. extract. Summarizing, higher activity of the mixture than the neat *Hypericum* L. and *Urtica* L. extract (towards Gram-positive cells) may be a confirmation of a synergistic effect between extracts. However, the reason of a high effectiveness of the mixture can also be high activity of the *Chelidonium* L. extract. Effectiveness of *Hypericum* L., and/or *Chelidonium* L. extracts against Gram-positive bacteria such as *S. aureus* was confirmed by many authors [8-12,14-16].

The preliminary analysis of antimicrobial activity of the mixture of described herbal extracts against *E. coli* strain revealed that 1% DMSO decreased the cells growth rate. However, it did not inhibit these microorganisms' growth completely. As was shown in Figure S2, LB medium with DMSO and herbal extracts mixture and with the neat extract of the *Chelidonium* L. inhibited *E. coli* cells. These findings confirmed that the *Chelidonium L, Urtica* L., neat extracts and extracts mixture were effective against Gram-negative bacteria. Effectiveness of *Hypericum* L., and/or *Chelidonium* L. and *Urtica* L. extracts against Gram-negative microorganisms such as *E. coli* was reported by many authors [8-12,14-16]. It should be mentioned that *Urtica L.* was confirmed to be effective mostly against Gram-negative bacteria. Analyzing the activity of

the *Hypericum* L. extract towards to *E. coli* strain, OD fall was observed only after 1 hour of incubation confirming that the activity of the mixture was higher than the effectiveness of this neat extract.

Analyzing the activity of neat *Chelidonium* L and *Urtica* L. extracts and the mixture of three (*Hypericum* L., *Chelidonium* L. and *Urtica* L.) extracts against both Gram-positive and Gram-negative microorganisms, it was observed that the initial OD was higher for neat extracts than for the mixture. The reason was the dark green color of neat *Chelidonium* L. and *Urtica* L. extracts (Fig. S1, S2) Additionally, an initial OD fall was higher for neat extracts (*Chelidonium L* cultivated with *S. aureus* and *Chelidonium* L. and *Urtica* L. cultivated with *E. coli*) than for the mixture of extracts. It was concluded that the higher, initial OD fall was caused by the mixing of neat extracts with LB medium (not by the higher activity if neat extracts than the activity of extracts mixture).

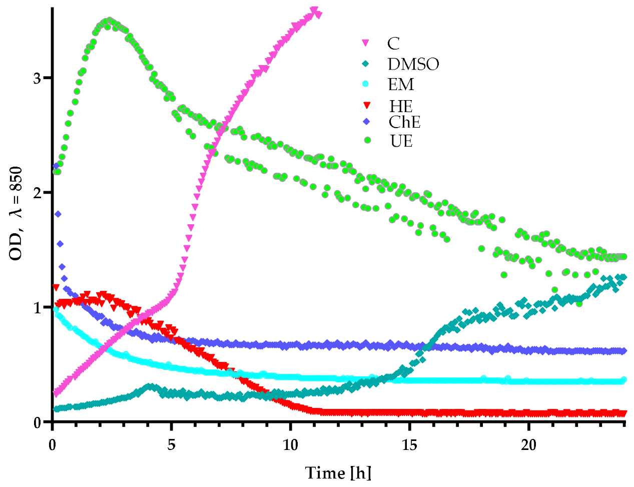

Figure S1. The growth of *S. aureus* in real-time in the presence of the mixture of *Hypericum* L. (HE), *Chelidonium* L. (ChE) and *Urtica L.* (UE) extracts, C- control.

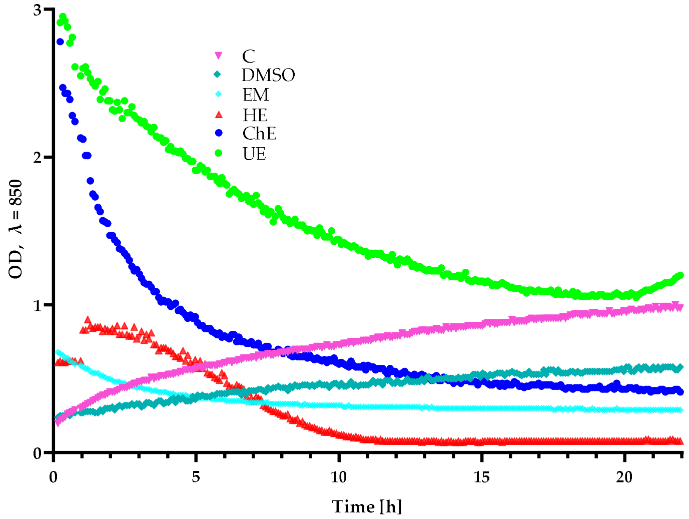

Figure S2. The growth of *E. coli* in real-time in the presence of mixture of *Hypericum* L. (HE), *Chelidonium* L. (ChE) and *Urtica L.* (UE) extracts, C-control.

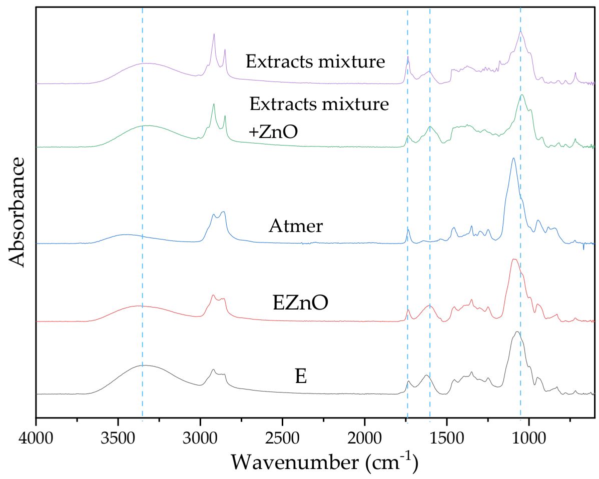

Figure S3. FTIR of additives (E, EZnO) and their individual components

Table S1. DSC results for the first and second run.

DSC first heating						
Sample acronym	Tg1	T_{g2}	T_{cc}	ΔH_{cc} [J/g]	T_m [μm]	ΔH_m [J/g]
PHA	-7.2	48.2	93.0	10.2	153.4	15.0
PHA-ZnO	-7.2	49.2	95.5	10.7	155.3	13.2
PHA-E	-7.3	48.7	94.3	11.0	154.3	15.9
PHA-EZnO	-7.5	45.9	92.4	11.6	154.5	14.2

DSC second heating							
Sample acronym	T_g	Tcc1	T_{cc2}	ΔH_{cc1} [J/g]	ΔH_{cc2} [J/g]	T_m [μm]	ΔH_m [J/g]
PHA	-8.2	69.1	98.6	11.8	11.2	154.5	27.7
PHA-ZnO	-7.9	73.4	97.5	9.6	8.8	155.9	26.9
PHA-E	-8.2	79.1	99.8	8.0	9.1	154.5	24.2
PHA-EZnO	-7.5	-	91.7	-	13.7	156.7	22.4

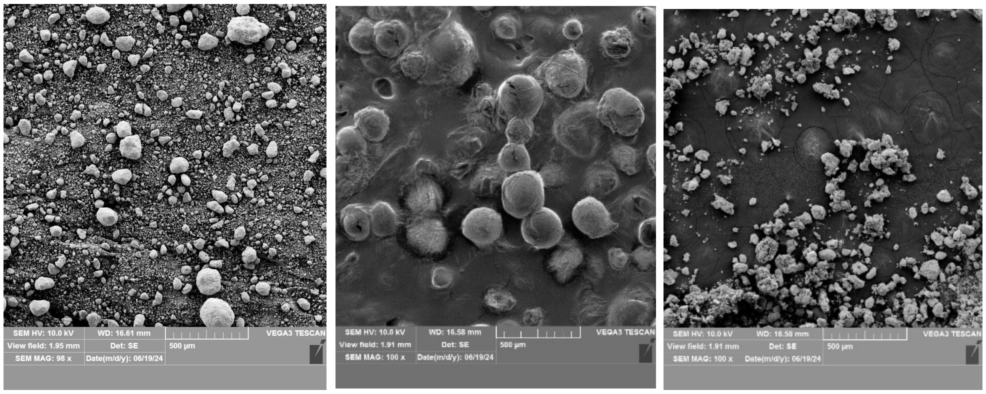

Figure S4. SEM micrographs of of ZnO (left), dried mixture of extracts (middle) and the mixture with ZnO (right) .

Table S2. MFI values for native granulate and regranulates

Sample acronym	MFI at 170 °C [g/10 min]
Native PHA*	18.5 (0.97)[c]
PHA	22.0 (2.80)[b,c]
PHA-ZnO	25.0 (1.51)[a,b]
PHA-E	22.1 (2.03)[b,c]
PHA-EZnO	26.5 (1.95)[a]

Table S3. TGA results

Sample acronym	T_{max} DTG $_{PHB}$ [°C]	T_{max} DTG $_{PLA}$ [°C]	T_{0d} [°C]
Native PHA*	-	-	-
PHA	269.4	331.7	257.1
PHA-ZnO	271.7	324.5	258.6
PHA-E	274.1	336.5	257.8
PHA-EZnO	280.0		261.2

Figure S5 shows FTIR-ATR spectra for all studied films. The set of characteristic bands confirmed that the main component of the bioresin is a blend of PHB and PLA and the PHB is a dominant fraction in the blend. Comparing different types of films, there were no differences between the spectra (no new bands and peaks' shifts appeared) observed. The lack of changes can be caused by the small amount of additives in the polymer matrix.

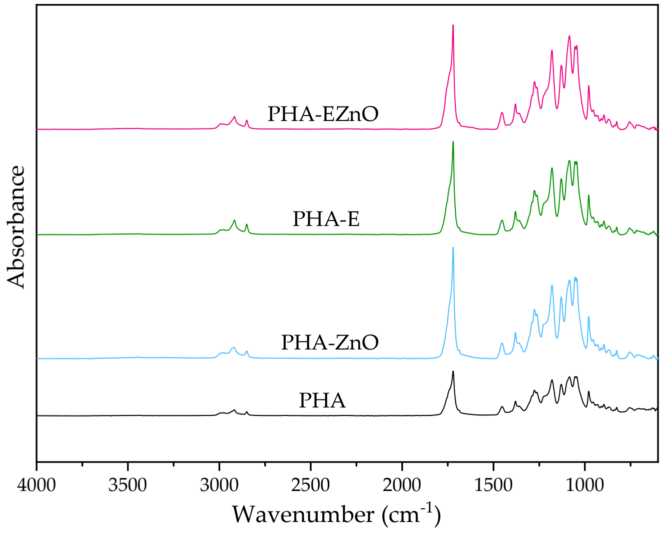

Figure S5. FITR-ATR spectra of PHA-based films.
